# Phylodynamics Helps to Evaluate the Impact of an HIV Prevention Intervention

**DOI:** 10.3390/v12040469

**Published:** 2020-04-20

**Authors:** Tetyana I. Vasylyeva, Alexander Zarebski, Pavlo Smyrnov, Leslie D. Williams, Ania Korobchuk, Mariia Liulchuk, Viktoriia Zadorozhna, Georgios Nikolopoulos, Dimitrios Paraskevis, John Schneider, Britt Skaathun, Angelos Hatzakis, Oliver G. Pybus, Samuel R. Friedman

**Affiliations:** 1 Department of Zoology, University of Oxford, OX1 3SY Oxford, UK; 2 New College, University of Oxford, OX1 3BN Oxford, UK; 3 Alliance for Public Health, Kyiv 03150, Ukraine; 4 Division of Community Health Sciences, University of Illinois at Chicago School of Public Health, Chicago, IL 60612, USA; 5 State Institution “The L.V. Gromashevsky Institute of Epidemiology and Infectious Diseases of NAMS of Ukraine”, Kyiv 03038, Ukraine; 6 Medical School, University of Cyprus, Nicosia 1678, Cyprus; 7 Department of Hygiene, Epidemiology and Medical Statistics, Medical School, National and Kapodistrian University of Athens, 157 72 Athens, Greece; 8 Department of Medicine, University of Chicago, Chicago, IL 60637, USA; 9 Department of Medicine, University of California San Diego, San Diego, CA 92093, USA; 10 Department of Population Health, New York University, New York, NY 10003, USA

**Keywords:** HIV, phylodynamics, birth-death model, prevention, intervention

## Abstract

Assessment of the long-term population-level effects of HIV interventions is an ongoing public health challenge. Following the implementation of a Transmission Reduction Intervention Project (TRIP) in Odessa, Ukraine, in 2013–2016, we obtained HIV *pol* gene sequences and used phylogenetics to identify HIV transmission clusters. We further applied the birth-death skyline model to the sequences from Odessa (*n* = 275) and Kyiv (*n* = 92) in order to estimate changes in the epidemic’s effective reproductive number (*R_e_*) and rate of becoming uninfectious (*δ*). We identified 12 transmission clusters in Odessa; phylogenetic clustering was correlated with younger age and higher average viral load at the time of sampling. Estimated *R_e_* were similar in Odessa and Kyiv before the initiation of TRIP; *R_e_* started to decline in 2013 and is now below *R_e_* = 1 in Odessa (*R_e_* = 0.4, 95%HPD 0.06–0.75), but not in Kyiv (*R_e_* = 2.3, 95%HPD 0.2–5.4). Similarly, estimates of *δ* increased in Odessa after the initiation of TRIP. Given that both cities shared the same HIV prevention programs in 2013–2019, apart from TRIP, the observed changes in transmission parameters are likely attributable to the TRIP intervention. We propose that molecular epidemiology analysis can be used as a post-intervention effectiveness assessment tool.

## 1. Introduction

In recent years phylodynamic analyses have become increasingly widely applied in the field of HIV surveillance and prevention. Phylodynamics can estimate changes in viral population size over time and be used to explore how various factors (epidemiological, evolutionary, behavioural, etc.) affect these changes [1]. Such analyses are often performed on sets of HIV genetic sequences collected through national or regional HIV drug resistance surveillance efforts. They can be used for identifying and monitoring of HIV transmission clusters, identification of local HIV outbreaks and for targeting subsequent public health interventions [2,3,4]. When the location from which the HIV genomes were obtained is known, phylogeographic methods can be used to describe HIV migration within various geographic regions [4,5,6], or among different epidemiological risk groups.

Phylodynamic models based on birth-death processes enable the estimation of three important epidemiological parameters: (i) the effective reproductive number, *R_e_*, which is the average number of secondary infections arising from an infected individual at any given time during an epidemic, (ii) the proportion of the population that has been sampled and (iii) the rate of becoming uninfectious, *δ,* which is the rate with which individuals cease transmitting infections (i.e., due to behavioural change, treatment or death) [7,8,9]. The effective reproductive number is often used to describe transmission dynamics in a population; *R_e_* > 1 means the epidemic is growing, while *R_e_* < 1 means the epidemic is declining. The becoming-uninfectious rate is inversely related to the average duration of the infectious period, assuming that length of this period is exponentially distributed. The becoming-uninfectious rate is important, because it is known that Treatment as Prevention (TasP) is most effective if people are diagnosed and treated as soon as possible after they are infected [10]. Thus, increasing the rate at which individuals cease to be infectious can reduce onward transmission and is clinically beneficial for the infected individual.

Over the last 10 years the scope of HIV prevention interventions has grown to incorporate new clinical insights and technological advances; it is now common for interventions to involve the collection of virus genetic sequences. Often these studies involve marginalised groups, such as people who inject drugs (PWID), men who have sex with men (MSM), female sex workers and undocumented migrants, as usually HIV prevalence is higher in these groups than in so-called “general population”. Importantly, it is often unrealistic to try to obtain a random sample from certain populations due to their hidden (and in some locations criminalized) nature, since a random sampling assumes that each individual is selected by chance and everyone has an equal chance to participate in the study. Social-network based approaches have proved to be an effective alternative to recruit participants for HIV research studies and to deliver HIV preventive measures in hard-to-reach populations [11]. The Transmission Reduction Intervention Project (TRIP) is one example of such a network-based intervention that was implemented in various settings and transmission groups from 2011 to 2016 [12,13]. TRIP aimed to evaluate a network-based approach to locating recently (within the last 6 months) HIV-infected people and to preventing ongoing transmission. Previously published results have shown that the TRIP intervention was more effective in finding recently HIV-infected people, those with high HIV viral load and those previously undiagnosed, than the standard of care options [13,14,15].

Odessa, Ukraine, was one of the first locations where TRIP was implemented. Ukraine has a large HIV-infected population (estimated to be 240,000 people as of 2019 [16]), but only 4 (out of 27 total) administrative regions account for >50% of HIV cases in the country: Odessa, Kyiv, Dnipro and Donetsk. Odessa is thought to be the “birthplace” of the HIV epidemic in Ukraine and the post-soviet territories [17], and it still has one of the highest HIV prevalence levels in various risk groups in Ukraine (i.e., 13% in MSM and 28% in PWID in 2015) [18,19]. Overall, Kyiv and Odessa share similar HIV epidemiological profiles and have experienced a similar number of HIV prevention interventions, other than TRIP. They were the first cities in which treatment started being offered to patients upon diagnosis in 2014, even though it is still not a universal practice in Ukraine. Since 2013, in both cities, a case management approach to linkage to care has been introduced for PWID: peer outreach workers help newly diagnosed HIV-positive PWID to register with local AIDS Centres and start antiretroviral treatment. During 2012–2013 the “Project Protect” intervention was implemented in both cities, which offered the PWID community education about the danger of acute HIV infection [20]. According to our previous estimates, Odessa and Kyiv were also some of the main sink (receiving infections) locations for HIV spatial dissemination associated with the recent military conflict in the east of Ukraine [21]. Neither city displayed a decline in the annual number of newly HIV diagnosed people in 2016–2019, while the annual number of performed HIV tests also grew [22].

It has been proposed that the analysis of HIV gene sequences can be used to assess the impact of a specific preventive intervention in a specific location [23]. Here we aimed to assess the population-level impact of TRIP on HIV transmission in Odessa, Ukraine, and to compare it to the estimated HIV transmission dynamics in the capital of Ukraine, Kyiv. We used a data set of HIV sequences from Odessa and Kyiv, Ukraine from 2000—2019, compiled from several sources. We then describe the application of phylodynamic methods to estimate the effective reproductive number and the becoming-uninfectious rate from these data, along with an analysis of HIV transmission clusters. We find that following the TRIP implementation in Odessa, the HIV epidemic there is characterized by a lower *R_e_* and a faster *δ* compared to Kyiv. Finally, we discuss the limitations and the potential of phylodynamic analysis to evaluate HIV prevention efforts.

## 2. Materials and Methods

### 2.1. Data

Our data set comprises HIV-1 subtype A *pol* genetic sequences (corresponding to HXB2 positions 2568–3256) compiled from three sources: (i) the TRIP intervention, (ii) AIDS Centres in Odessa and Kyiv and (iii) the Los Alamos National Laboratory HIV sequence database, LANL (https://www.hiv.lanl.gov). This includes 161 newly generated sequences from the TRIP intervention conducted in Odessa in 2013–2016. In TRIP, people were screened for HIV and, if positive, further tested to determine whether the infection was recent (defined by a Limiting Antigen avidity test or by a negative HIV test result within the previous 6 months). Those with recent HIV infection were invited to participate in TRIP and were given coupons to invite their sexual and injecting partners into the intervention. Coupons were also given to their partners, but then the recruitment stopped unless another recently HIV infected person was found in their network. Thus, within the TRIP data, we differentiated between samples that (a) came from people screened for recent infection who were not recently infected, and thus were not invited to further participate in the network intervention component of TRIP (Non-participant TRIP samples, NPTS) and samples which (b) came from TRIP participants—either recently infected people or their 1st or 2nd degree network contacts (participant TRIP samples, PTS). Further details on the TRIP data collection and genetic sequencing procedures can be found in the original publications [13,24]. We obtained 197 sequences of the subtype A *pol* gene (84 newly generated) from patients of AIDS Centres (HIV treatment clinics) in Odessa (*n* = 105) and Kyiv (*n* = 84) collected in 2012–2019. These samples were sequenced at the L.V.Gromashevskij Institute of Epidemiology and Infectious Diseases in Kyiv using ViroSeq^®^ HIV-1 Genotyping System v2.0 Kit (Celera Corporation, Abbott Laboratories, Abbott Park, IL, USA) [25]. Finally, we downloaded from the LANL database all publicly available HIV-1 subtype A *pol* sequences that (i) corresponded to the same genomic region, (ii) were sampled from Odessa (*n* = 25) and Kyiv (*n* = 12) and had available sampling year and city information. See the Table S1 for accession numbers of the sequences used in the analysis. Accession numbers for TRIP sequences are MT348782–MT348931.

We further grouped the sequences into the following datasets: *Odessa* and *Kyiv* datasets comprised sequences from Odessa and Kyiv from LANL and AIDS Centres; sequences from TRIP were added to the *Odessa* dataset. Duplicates in both datasets were removed using the ElimDupes tool from the LANL website (https://www.hiv.lanl.gov/). Additionally, most PTS sequences had information about the participant’s transmission risk group, HIV viral load, HIV recency status (as defined by the TRIP intervention protocol) and information on co-infection with hepatitis C virus (HCV), hepatitis B virus (HBV) and syphilis (co-infections determined by a rapid multi-test).

All TRIP participants gave informed consent under protocols approved by the IRBs of the National Development and Research Institutes, USA, and the medical ethics committee at L.V. Gromashevsky Institute of Epidemiology and Infectious Diseases, Ukraine.

### 2.2. Phylogenetic Analysis

We used the REGAv3 [26] and COMET [27] subtyping tools to confirm that the sequences’ subtypes were correctly labelled. We estimated maximum likelihood (ML) phylogenetic trees for both datasets using RAxML [28]. In all of the ML analyses we used an HKY nucleotide substitution model with gamma-distributed rate variation among sites and ran a bootstrap analysis with 100 replicates. We identified potential transmission clusters in the Odessa phylogenetic tree with ClusterPicker [29]. Any clade with two or more sequences, within-cluster genetic distance <1.5%, and bootstrap statistical support >90% was defined as a possible transmission cluster, in accordance with the literature [30]. To study differences between those individuals who were placed in clusters and those who were not, we used t-tests (for continuous variables) and Fisher’s exact test (for categorical variables) to compare socio-demographic characteristics.

### 2.3. Phylodynamics and Estimation of Epidemiological Parameters

We first reconstructed time-calibrated phylogenetic trees from the Odessa dataset and estimated the time to the most recent common ancestors (TMRCA) of all identified transmission clusters using BEASTv1.10.4 [31]. First, we used path-sampling [32] and stepping stone [33] marginal likelihood estimators (MLE) to select the most appropriate coalescent model. The non-parametric Bayesian skyline plot (BSP) model [34] was selected over the constant population size and exponential growth models (see Table S2) and was therefore used in further analyses. For the subsequent BSP analyses, we used an HKY nucleotide substitution model with gamma-distributed rate variation among sites (HKY+G). Given the poor molecular clock signal in our dataset (as assessed using TempEst [35]), we relied on previously published estimates of the evolutionary rate to calibrate the molecular clock. Specifically, we used a lognormal relaxed molecular clock model with a continuous-time Markov chain (CTMC) prior on the mean (constrained to values between 1 × 10^−3^ – 3 × 10^−3^) and a normal prior on the standard deviation (mean = 5 × 10^−4^, standard deviation = 5 × 10^−4^), as previously reported in [36,37]. We also used a normal distribution prior for the TMRCA of the tree (mean = 35, standard deviation = 5). We used the same nucleotide substitution and molecular clock models for all subsequent phylodynamic analyses. Markov chain Monte Carlo (MCMC) sampling was performed for 300 × 10^6^ iterations (with 20% removed as burn-in). We used Tracer [38] to visually inspect the convergence of the MCMC runs for all BEAST analyses and to ensure a minimum effective samples size of 200, as is standard practice [4,39].

**Figure 1:**
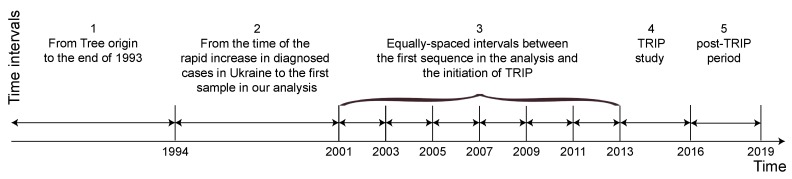
Time intervals specified for the birth-death skyline model (BDSKY) analysis.

We further applied a birth-death skyline model (BDSKY) [8] implemented in BEASTv2.6.0 [40] to estimate *R_e_* and the becoming uninfectious rate (*δ*) for *Odessa* and *Kyiv* independently. The BDSKY model assumes that *R_e_* and *δ* are piecewise constant functions through time; for this analysis we specified 10 time-intervals (Figure 1) that correspond to:

(1) the time between the origin of the tree and 1993 (N of sequences = 0 in both Odessa and Kyiv);

(2) the time between 1994, which corresponds to the year when the number of newly diagnosed HIV cases dramatically increased in Ukraine [41,42]), and the first sequence in the analysis (N of sequences = 0 in both Odessa and Kyiv);

(3–8) multiple intervals of equal duration between the collection date of the first sequence in the analysis (2001 for Odessa, and 2000 for Kyiv) and the initiation of TRIP in 2013 (N of sequences = 22, 0, 0, 0, 3, 1 in Odessa and *n* = 2, 0, 1, 0, 9, 3 in Kyiv in 2001–2002, 2003–2004, 2005–2006, 2007–2008, 2009–2010, 2011–2012, correspondingly);

(9) the time period during which the TRIP intervention has been implemented, corresponding to the date of sampling of the first and the last TRIP sequence (June 2013–February 2016) (N of sequences = 205 in Odessa and *n* = 54 in Kyiv);

(10) the time after the TRIP intervention ended, corresponding to the time interval between the sampling date of the youngest TRIP sequence and the sampling date of the youngest sequence in the dataset (February 2016–February 2019) (N of sequences = 44 in Odessa and *n* = 23 in Kyiv).

The BDSKY model assumes individuals become non-infectious upon sampling (i.e., they cannot transmit HIV further) as they are provided treatment and reach viral suppression. The prior distributions for the BDSKY model consisted of: (a) a uniform distribution for the date of origin, which is the duration of the epidemic represented by the sequences in the analysis, with an upper bound of 60 and lower bound 19 (the age of the oldest sequence in the analysis); (b) a lognormal distribution for *R_e_* (mean = 1.0, standard deviation = 1.25) with an upper bound of 10; (c) a lognormal distribution for *δ* (mean = 0.0, standard deviation = 1.0) constrained to take values between 0.05 - 6, corresponding to the plausible values of the duration of infectious period (20 years–2 months); and (d) a beta prior for the sampling proportion (α = 1.0, β = 10.0, constrained to values between 0 and 0.01) for time intervals (3)–(10) as defined above; the sampling proportion was set to 0 before the first sampling date (time intervals (1) and (2) as defined above). We ran 4 independent MCMC chains for 300 × 10^6^ generations (with the initial 3% removed as burn-in) for the *Odessa* dataset. For the analysis of the *Kyiv* dataset we used the same analyses specifications and ran 2 independent MCMC analyses for 300 × 10^6^ generations (10% burn-in). We also used the same BEAST specifications in our consequent sensitivity analyses. We used LogCombiner [40] to combine results from multiple chains.

XML files specifying all the analyses and data described above are available at the GitHub repository https://github.com/HIVMolEpi/TRIP_Ukraine.

## 3. Results

### 3.1. Data and Phylogenetic Clustering

Between 2013–2016, 1252 participants were screened for recent HIV infection as part of the TRIP project in Odessa, Ukraine. For 67 non-recently infected participants screened at TRIP an HIV sequence was generated (NPTS sequences). Forty individuals were identified as recently infected according to the TRIP definition of “recency” [13], and a further 369 HIV-positive individuals were recruited from their networks [15], these individuals contributed an additional 94 HIV *pol* sequences (the PTS sequences). After removing duplicates and non-subtype A sequences, 65 NPTS, 85 PTS, 25 LANL and 100 sequences available from the Odessa AIDS Centre were included in the *Odessa* dataset, giving a total of 275 sequences collected from 2001–2019. Table 1 reports the socio-demographic and clinical characteristics of the individuals from whom NPTS and PTS sequences were obtained (*n* = 150).

**Figure 2:**
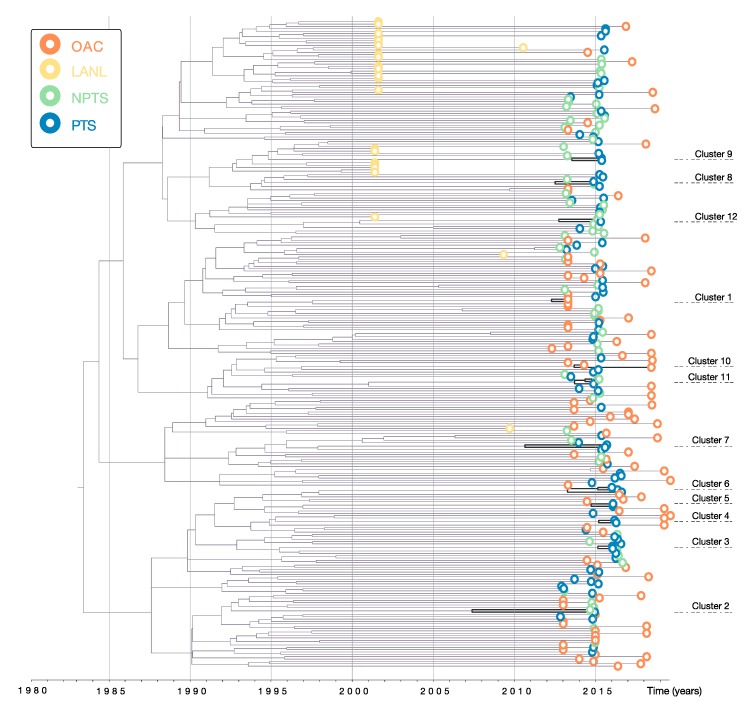
Molecular clock tree reconstructed from sequences in *Odessa* dataset. The thick black lines indicate the identified probable transmission clusters. OAC – sequences from the Odessa AIDS Centre patients, LANL – sequences from the Los Alamos National Laboratory HIV sequence database, NPTS - Non-participant TRIP samples, PTS - participant TRIP sample.

Based on the ML phylogenetic tree reconstructed from the *Odessa* dataset (See Figure S1), we identified 12 probable transmission clusters: 10 pairs and 2 clusters of three sequences. These clusters also had high posterior probability (>0.9) support on the maximum clade credibility tree reconstructed in the BSP analysis (Figure 2).

Clusters 1 and 10 consisted of sequences isolated from AIDS Centre patients, thus no additional information was available for these sequences (e.g., age and HCV status). The sequences in the remaining ten clusters came from TRIP. The individuals from whom these sequences were obtained were younger on average than their unclustered counterparts (t-test *p*-value = 0.05, H_0_ = both groups have the same average age) and had approximately twice their viral load (*p*-value = 0.04, H_0_ = both groups have the same average viral load) (Table 1). However, these associations were not statistically significant after adjustment for multiple comparisons using the false discovery rate method (adjusted t-test *p*-value = 0.25 for age and *p*-value = 0.25 for viral load) [43].

Most of the sequences in the clusters came from individuals that (a) share a route of transmission (apart from clusters 2, 7 and 9); (b) were HCV-positive; (c) were HBV negative; (d) did not have syphilis (apart from clusters 6 and 11); and (e) were male (Table 2). Half of the clusters included at least one recently infected individual. Two clusters, 6 and 11, included individuals co-infected with syphilis, suggesting a possible sexual transmission route.

After removing duplicates and non-subtype A sequences, the *Kyiv* dataset comprised 92 sequences, those sampled at the AIDS Centres and available through the LANL database. The ML phylogenetic tree reconstructed from Kyiv sequences is presented in Figure S2. Using our definition of phylogenetic clusters, no clusters were found on the tree reconstructed from the *Kyiv* dataset.

### 3.2. BDSKY Analysis

**Figure 3:**
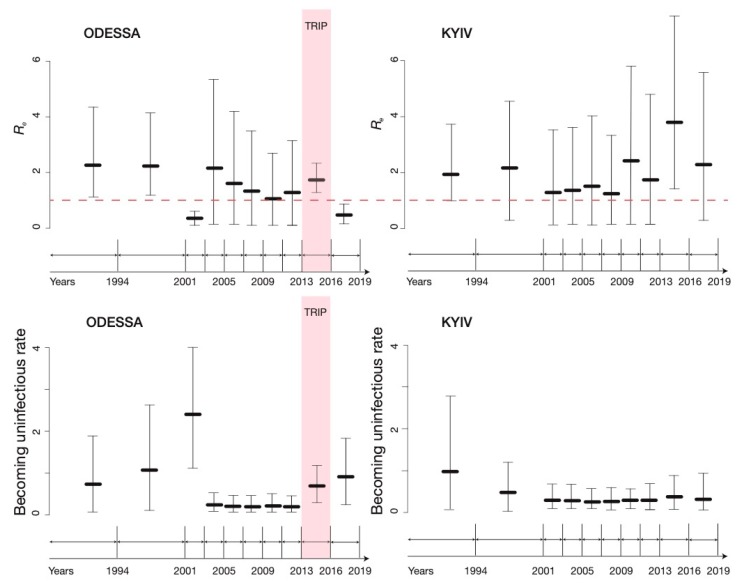
Estimates of the effective reproductive number, *R_e_*, in Odessa and Kyiv (top), where the red dotted line represents the epidemiological threshold of *R_e_* =1; and the becoming uninfectious rate (bottom) obtained from the *Odessa* and *Kyiv* datasets by means of the BDSKY analysis.

The BDSKY analysis of the *Odessa* dataset showed that from the beginning of the epidemic and until early 2016 the *R_e_* in Odessa remained consistently above 1 with the exception of 2001–2002 when it dropped to *R_e_* = 0.27 (95% Highest Posterior Density (HPD) 0.04–0.52, Figure 3). With the exception of 2001–2002, there was little difference in *R_e_* in Odessa and Kyiv before the TRIP intervention: in 2011–2012 the *R_e_* was estimated to be 1.19 (95% HPD 0.03–3.07) in Odessa and 1.66 (95% HPD 0.04–4.7) in Kyiv (Figure 3). In 2013–early 2016, during the implementation of TRIP *R_e_* was estimated to be 1.66 (95% HPD 1.19–2.2) in Odessa and *R_e_* = 3.7 (95% HPD 1.32–7.5) in Kyiv. After TRIP, in 2016–2019, it declined to below the epidemiological threshold of 1 necessary to maintain growth of the epidemic in Odessa (*R_e_* =0.4, 95% HPD 0.06–0.75). During the same time *R_e_* has likely remained high in Kyiv, estimated to be 2.32 (95% HPD 0.2–5.4).

The becoming-uninfectious rate for Odessa grew between the mid-1980s and 2000 from 0.7 before 1994 (corresponding to an infectious period of 1.4 years) to 2.65 in 2001–2002 (corresponding to infectious period of 5 months (Figure 3). This rate then dropped substantially to 0.2 in 2003–2004 (corresponding to an infectious period of 5 years) and remained low until the beginning of the TRIP project in 2013. In 2013–early 2016 the becoming-uninfectious rate was estimated to be 0.72 (corresponding to an average infectious period of 1.4 years) and it continued to grow after the end of TRIP to be 0.94 (corresponding to an average infectious period of 1.1 years) in 2016–2019. Analysis of the *Kyiv* dataset revealed that the becoming-uninfectious rate has gradually decreased over time, from 0.97 (corresponding to an average infectious period of 1 year) before 1994 to 0.33 (an average infectious period of 3 years) in 2016–2019 (Figure 3. Importantly, no difference in the becoming-uninfectious rate between Odessa and Kyiv was observed for 10 years before the TRIP intervention (2003–2013), when the average duration of infectious period in Odessa started to decline.

After the initial epidemic growth period in late-90s/early 2000s, the BDSKY did not provide precise estimates for *R_e_* or detect any changes in the becoming uninfectious rate for the period when no or very few sampling events occurred (2002–2012). For the duration of this period, both locations had an *R_e_* estimate just above 1 with confidence intervals from 0 to 4. Similarly, in both Odessa and Kyiv the becoming uninfectious rate stabilized at around 0.2 between 2003–2013, corresponding to an average infectious period of 5 years.

### 3.3. Sensitivity

**Figure 4:**
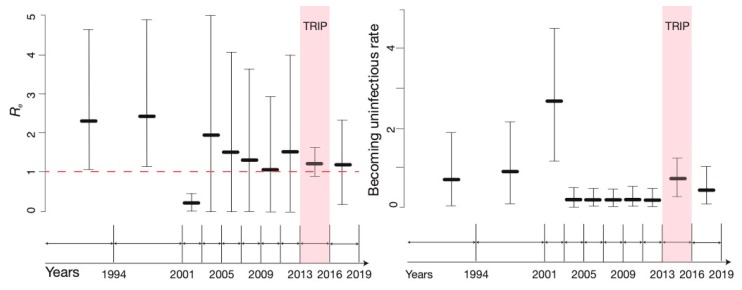
Temporal estimates of the effective reproductive number, *R_e_*, and the becoming uninfectious rate, obtained from *Odessa* datasets after removal of all sequences generated by network-based recruitment of TRIP participants. The red dotted line represents the epidemiological threshold of *R_e_* =1.

We performed several sensitivity analyses to explore the robustness of our estimates. First, we used a less strict definition of clusters for ClusterPicker analysis (bootstrap >80%, using the same tree and number of bootstrap replicates as for the analysis with higher bootstrap cut-off value), but this resulted in only one additional cluster. Second, we removed 65 PTS sequences generated by network-based recruitment in TRIP from the *Odessa* dataset, keeping the 65 NPTS sequences and 20 PTS sequences obtained from recently infected individuals at screening (thus, not obtained through network recruitment) in the analysis. This resulted in a dataset of *n* = 210. The ML phylogenetic tree reconstructed from this dataset can be found in Figure S3. After the network-derived sequences were eliminated, only 4 phylogenetic clusters were left on the Odessa phylogenetic tree, which presented a more similar figure to that in Kyiv, where no transmission clusters were identified. We then reran the BDSKY analysis with this dataset to make sure that no bias was introduced by these potentially-correlated samples (Figure 4).

This analysis yielded a lower mean estimate of *R_e_*, 1.23 (95% HPD 0.9–1.64), during the implementation of the TRIP in Odessa in 2013–early 2016, and a larger mean estimate, *R_e_* = 1.2 (95% HPD 0.2–2.33), for the period after the TRIP intervention, in 2016–2019. Importantly, the *R_e_* estimates in Odessa since the initiation of TRIP in 2013 remained substantially lower than the corresponding estimates for Kyiv, though the difference between the two estimates was no longer significant in 2016–2019. For the becoming uninfectious rate in this analysis, the estimate was the same for the duration of TRIP as when the full dataset was analysed. For the years after the intervention (2016–2019), the becoming uninfectious rate was lower, at 0.4, corresponding to an average infectious period of 2.4 years for Odessa, which was only a little higher than our estimates in Kyiv of 0.33, corresponding to an average infectious period of 3 years.

Finally, to make sure that our findings cannot be simply explained by a larger number of sequences in *Odessa* dataset compared to *Kyiv* dataset (*n* = 275 vs. *n* = 92), we have rerun the BDSKY analysis with 92 randomly selected sequences from the *Odessa* dataset. The mean estimates and 95% HPD obtained from this analysis did not substantially differ from the estimates obtained in the analysis of the full dataset (Figure S4).

## 4. Discussion

In the last five years, the amount of virus genetic data available for phylogenetic and phylodynamic analysis has substantially increased; in Ukraine alone, the number of HIV genetic sequences has quadrupled since 2018. This presents new opportunities to apply phylogenetic and phylodynamic techniques to describe changes in virus populations and in HIV transmission dynamics in the country. Here, we examined factors associated with HIV phylogenetic clustering and applied a birth-death phylodynamic model to examine potential differences in HIV transmission dynamics between two cities (Odessa and Kyiv), following the introduction in one city (Odessa) of an HIV prevention intervention (TRIP). We found a reduction in the HIV effective reproduction number (i.e., reduced epidemic growth) in Odessa relative to Kyiv, where no TRIP intervention was enacted.

We have identified and described HIV genetic clustering patterns, as these are known to be informative for public health planning [44]. Interestingly, we did not observe many of the clustering patterns reported in other settings. Specifically, in our study, clustering status was not associated with gender, risk group or recent HIV infection. The latter is particularly surprising given that network contacts of recently-infected people were actively recruited in TRIP, and given that multiple previous reports have found that recently-infected individuals are more likely to be included in genetic clusters [45,46,47]. Moreover, modelling studies have showed that such clustering can be expected and is explained by intensive transmission during the early stages of HIV infection [48]. Absence of such an association in our analysis can be explained potentially by the low number of observed clusters. Clustering was associated with higher viral load in our sample (though this association lost significance after multiple test correction), which is similar to the findings from the TRIP intervention undertaken in Athens and can be interpreted as a marker of recent infection [24], but could also be explained by other factors (e.g., co-infections, late-stage HIV infection). Being in a phylogenetic cluster in our study was correlated with younger age, which might be a proxy for more recent transmission events; this is similar to the result of a previous report from Belgium [49], but unlike the results reported for some other European countries and Canada [50]. Unfortunately, we were not able to differentiate between heterosexual and MSM transmission in our analysis due to the way the data were collected and all sexually-acquired infections were analysed together. This prevents us from making direct comparisons with other studies that showed that MSM infections are more likely to cluster compared to infections acquired through other transmission routes [46,49].

The additional meta-data associated with the sequences in our study helped us to question some of the more obvious clustering patterns. For example, a co-infection with syphilis might suggest a possible sexual route for HIV transmission, or if clustered sequences came from PWID then a parenteral route of transmission might be suspected, as in cluster 6 in our analysis. In some cases, combining information about recent infection and estimates of TMRCA allowed us to rule out the potential for direct transmission between individuals whose sequences were clustered. For instance, a recently-infected individual in cluster 2 was recruited in 2016, which means that individual could not have been infected earlier than 2015, while the TMRCA of cluster 2 is much older (2008.0, 95% HPD 2003.1–2012.9) precluding direct transmission within this cluster. Similarly, in cluster 7, a recently infected individual was recruited in 2016 while the TMRCA of the cluster was estimated to be 2010.7 (95% HPD 2007.0–2014.0).

Our analysis of changes in transmission dynamics in Odessa following the TRIP intervention suggested that epidemic growth rate was reduced relative to that in Kyiv during the same period and that the beginning of the TRIP intervention in Odessa coincides with the slowing down of HIV transmission in that city. We observe that estimates of the reproductive number decreased and estimates of the rate with which people become diagnosed (and within the assumption of our model, treated) increase. Crucially, no such trend was observed in Kyiv during the same time. This difference might be attributed to the fact that TRIP incorporated active network recruitment as part of its procedures and was able to reach to comparatively high number of people with undiagnosed HIV infection [14]. Thus, large networks of people were screened and linked to care in Odessa, while also raising awareness in the community. This could have led to the changes in *R_e_* and the becoming-uninfectious rate, both during and after the implementation of TRIP.

Our estimates of *R_e_* both in Odessa and Kyiv prior to the initiation of TRIP are different from previously reported estimates [42,51]. For the mid-90s, our BDSKY estimates (*R_e_* = 2.08, 95%HPD 0.21–4.41, for Kyiv and *R_e_* = 2.17, 95%HPD 1.11–4.07, for Odessa) are much lower than the estimates of the basic reproductive number that we have previously obtained through a coalescent-based analysis of HIV-1 subtype A *env* sequences from Russia and Ukraine (*R_e_* = 7) [42]. Even higher *R_e_* estimates were obtained through other birth-death models applied to the same *env* sequences (sampled in 1996–2011) by Magee et al. [51]. For the mid-2000s our estimates are relatively high, though this is accompanied by a large degree of uncertainty, i.e., large HPD intervals, which means that the true value might be included in the broad confidence intervals and is not very different from the previous reports that suggest that *R_e_* in Ukraine was below 1 after 2000 [51]. However, we believe that these discrepancies are largely due to the different number and sampling dates of sequences in the analysis. As suggested by Magee et al. [51], when no birth events (i.e., “infection events”) were observed in the tree during the 2000s, a very low *R_e_* was inferred. Similarly, in our analysis the *R_e_* in Odessa was estimated to be very low in 2001–2002, probably attributed to the absence of sampled sequences and birth events in the years after (2003–2009). In our analysis, the majority of sequences were sampled after 2011 and multiple birth events were observed in the tree during mid-2000s, which resulted in higher *R_e_* estimates after 2002. The broad confidence intervals in our analysis are not surprising, as it has been reported before that the BDSKY approach returns particularly imprecise estimates if the analysis goes further into the past than the oldest sampling date in the analysis [19,50]. However, as we are primarily interested in the dynamics about the time of the TRIP intervention, the broad HPDs at different time periods are of lesser concern. In the recent years, the HPDs were wider in Kyiv compared to Odessa, however our sensitivity analysis showed that it is unlikely to be attributed solely to the lower number of sequences from Kyiv, as our reduced *Odessa* dataset resulted in the values and HPDs comparable to those obtained from the full *Odessa* dataset.

Importantly, we analysed sequences from Kyiv and Odessa separately as two unrelated epidemics, even though they do not form two independent clades on the Ukrainian HIV phylogenetic tree. Given that the two epidemics share the same ancestry and, thus, parts of the phylogenetic tree, we might have introduced bias for our estimates that go further in the past. In this analysis, apart from 2001–2003, our estimates for both *R_e_* and the becoming uninfectious rate are very similar for Odessa and Kyiv for the period before TRIP. While it is not crucial for the time periods during and after the TRIP intervention, which are relevant in our analysis, this might to some extent explain why our *R_e_* estimates differ from other studies that focused on the Ukrainian HIV-1 epidemic in mid-90s–2000s.

We want to emphasise that currently there is no way to account for non-random sampling approaches in a phylodynamic context and we do not know how network-based sampling might be affecting estimates obtained in phylodynamic analyses of sequence data, which presents a particular theoretical challenge for the molecular epidemiology field [1]. In the context of HIV transmission, as well as many other infectious diseases, including viral hepatitises, a random sample of an infected population is often unrealistic and epidemiologists proceed with non-probability sampling methods. Consequently, more research is needed to develop phylodynamic frameworks that would be able to account for and accommodate these sampling techniques. Indeed, the sampling approach employed in this work might have biased some of our estimates. The fact that TRIP was a social network-based intervention, with an active contact recruitment approach means that some potential transmission events might be captured in our tree, that otherwise would not be captured under random population sampling. Specifically, the tree may be enriched for infection events among individuals with close or direct epidemiological linkage. It is possible that this would only bias our estimates towards a higher *R_e_* in Odessa, as *observing* more recent transmission events would be misinterpreted as increased transmission by the model. This would particularly strongly affect any analyses carried out in a coalescent framework, while birth-death models implemented here provide slightly more control through the sampling parameter. Nevertheless, we undertook a sensitivity analysis to investigate this concern. Removing any network-derived TRIP sequences, we still found a reduction in *R_e_* in Odessa since 2013 relative to that in Kyiv (Figure 3 and Figure 4), and an even more substantial reduction for the period when the TRIP intervention was implemented.

Crucially, if better surveillance data were available, it could be used to inform priors on some of the birth-death model parameters—particularly on the sampling proportion parameter—which could result in a significant reduction in the uncertainty of other parameters in the analysis. However, this could only be useful when reliable surveillance data are available and would need to be implemented with caution, as we have previously argued that if sampling assumptions of the model are violated, then discrepancy between the observed sampling times and the sampling priors can lead to biased *R_e_* estimates [52].

The sensitive nature of the questions in the study precluded us from obtaining personal identification information at the point of recruitment. This could have led to individuals appearing multiple times in the dataset, which would introduce biases. ElimDupes was used in an attempt to ameliorate potential repeated recruitment. For example, cluster 3 includes 2 sequences isolated from individuals of the same gender, age, risk group and very similar viral load. Thus, we cannot fully rule out the possibility that cluster 3 includes 2 subsequently sampled strains from one individual, seen by different interviewers. Our sensitivity analyses suggest that if that was the case, and we removed cluster 3 from our analysis, we have found that no statistically significant difference in average viral load between clustered and unclustered individuals would be observed. However, using ElimDupes to ensure that no patient was represented twice in our sample might have reduced our power to detect phylogenetic clusters, as it might happen that two patients have identical sequences.

## 5. Conclusions

The use of HIV genetic sequences allowed us to investigate the impact that an intervention might have had on a specific community and epidemic dynamics, during and after the years it was implemented. While there might have been other factors that have contributed to the reduction in *R_e_* and the decrease in the average infectious period in Odessa, this exercise allowed us to monitor transmission dynamics in certain communities and further promote preventive strategies associated with a positive dynamics in monitored epidemiological parameters. Importantly, molecular epidemiology methods are a useful tool when investigating the effect of certain prevention strategies and should be widely applied in public health settings.

## Figures and Tables

**Table 1:** Characteristics associated with the HIV sequences collected within the Transmission Reduction Intervention Project (TRIP) intervention.

Variables	Total (*n* = 150)	Clustered (*n* = 22)	Non- clustered (*n* = 128)	Fisher’s exact test/*t*-test *p*-value ***
Age (mean)	35.1	31.6	36.9	**0.05**
Women (N, % *)	46 (33.1)	6 (27.3)	40 (34.5)	0.63
PWID (N, %)	52 (40.6)	12 (54.5)	40 (37.7)	0.16
Years since 1st injection (for PWID, mean)	13.3	11.5	15.12	0.28
Recently infected (N, %)	28 (18.7)	6 (27.3)	22 (17.3)	0.37
HIV Viral load (mean)	142,740.6	245,157.6	113,935.8	**0.04**
HCV co-infection (N, %)	61 (53.5)	14 (63.6)	47 (51.1)	0.35
HBV co-infection (N, %)	5 (3.9)	0	5 (4.9)	0.59
Syphilis co-infection (N, %)	15 (11.6)	3 (13.6)	12 (11.2)	0.72
Index participants ** (N, %)	84 (56.4)	9 (40.9)	75 (59.1)	0.16

* % excludes missing values. ** Participants NOT recruited through coupons. *** values marked in bold reached statistical significance at 0.05 level. Fisher’s exact test was used to compare groups within variables.

**Table 2:** Potential transmission clusters identified in Odessa.

Cluster	N	Year of Cluster Origin		Transm. Route	HCV	HBV	Syph-ilis	Includes Recently Infected Person	Gender	TRIP Recruit-ment Group
		Year	95% HPD							
1	2	2012.7	2010.8–2014.0	Unk	Unk	Unk	Unk	Unk	Unk	N/A
2	2	2008.0	2003.1–2012.9	Drug use/sexual	Pos	Neg	Neg	Yes	Male	PTS/NPTS
3	2	2014.5	2013.0–2015.6	Drug use	Pos	Neg	Neg	Yes	Male	PTS
4	2	2014.5	2012.9–2015.7	Drug use	Neg	Neg	Neg	No	Male	PTS
5	2	2014.1	2012.2–2015.5	Drug use	Pos	Neg	Neg	No	Male	PTS
6	3	2012.5	2009.7–2014.9	Drug use	Pos	Neg	Neg/ Pos	No	Male/Female	PTS
7	2	2010.7	2007.0–2014.0	Drug use/sexual	Neg	Neg	Neg	Yes	Male/Female	PTS
8	2	2012.9	2010.3–2015.1	Sexual	Neg	Neg	Neg	Yes	Male/Female	PTS
9	2	2013.9	2011.4–2016.0	Drug use/sexual	Pos	Neg	Neg	No	Male	PTS
10	2	2014.1	2012.4–2015.0	Unk	Unk	Unk	Unk	Unk	Unk	N/A
11	3	2014.2	2012.5–2015.5	Sexual	Pos	Neg	Neg/ Pos	Yes	Female	PTS/NPTS
12	2	2013.1	2010.3–2015.3	Sexual	Neg	Neg	Neg	No	Male	PTS/NPTS

Unk—unknown, PTS—participant TRIP samples, NPTS—non -participants TRIP samples.

## Supplementary Materials

The following are available online at https://www.mdpi.com/1999-4915/12/4/469/s1, Figure S1: Maximum likelihood phylogeny estimated from the Odessa dataset, mid-point rooting, Figure S2: Maximum likelihood phylogeny estimated from the Kyiv dataset, mid-point routing, Figure S3: Maximum likelihood phylogeny estimated from the Odessa dataset after removing the network-derived sequences, mid-point rooting. Remaining potential transmission clusters are marked in yellow, Figure S4: Temporal estimates of the effective reproductive number, *R_e_*, and the becoming uninfectious rate, obtained from the reduced (N = 92) Odessa dataset, Table S1: Sequence accession numbers, Table S2: Model selection procedure with the path-sampling (PS) and stepping-stone (SS) marginal likelihood estimators (MLE).
