# Supplementary material for: Phylodynamics Helps to Evaluate the Impact of an HIV Prevention Intervention
Source: Viruses. 2020 Apr 20;12(4):469. doi: 10.3390/v12040469 (PMC7232463; doi:10.3390/v12040469)
Supplement: Supplementary file 1 [file viruses-12-00469-s001.pdf]

viruses

MDPI

Supplementary Materials

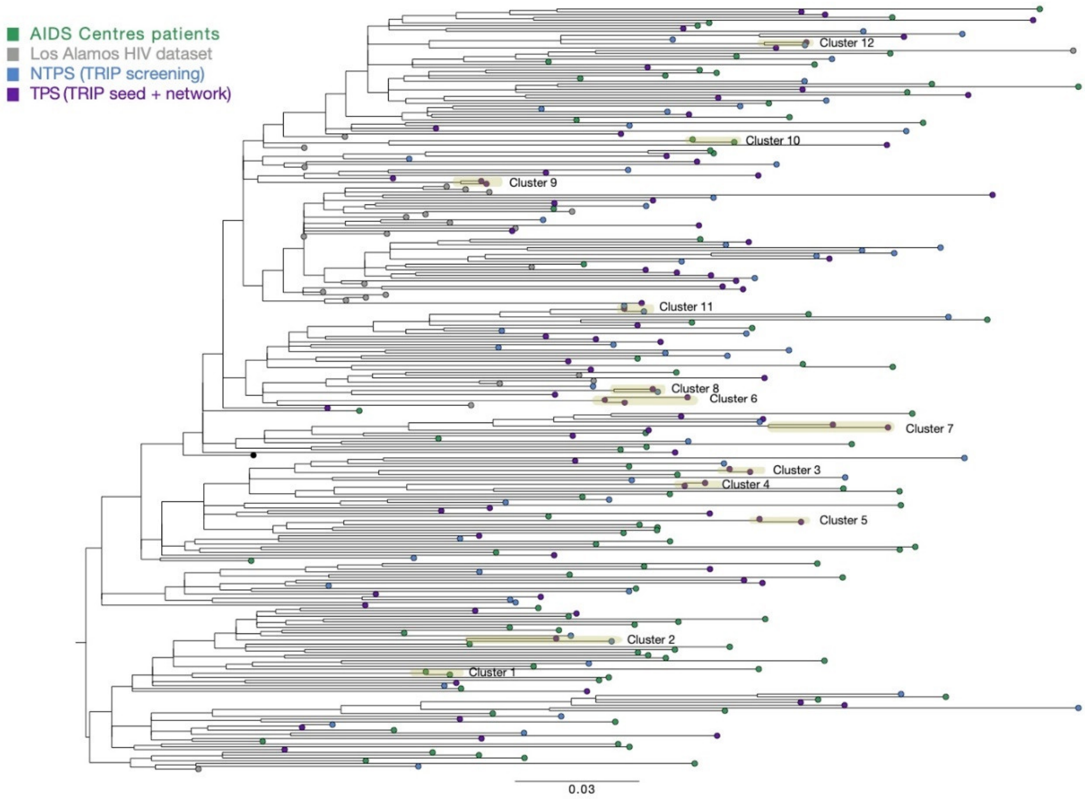

Figure S1. Maximum likelihood phylogeny estimated from the *Odessa* dataset, mid-point rooting. Potential transmission clusters are marked in yellow.

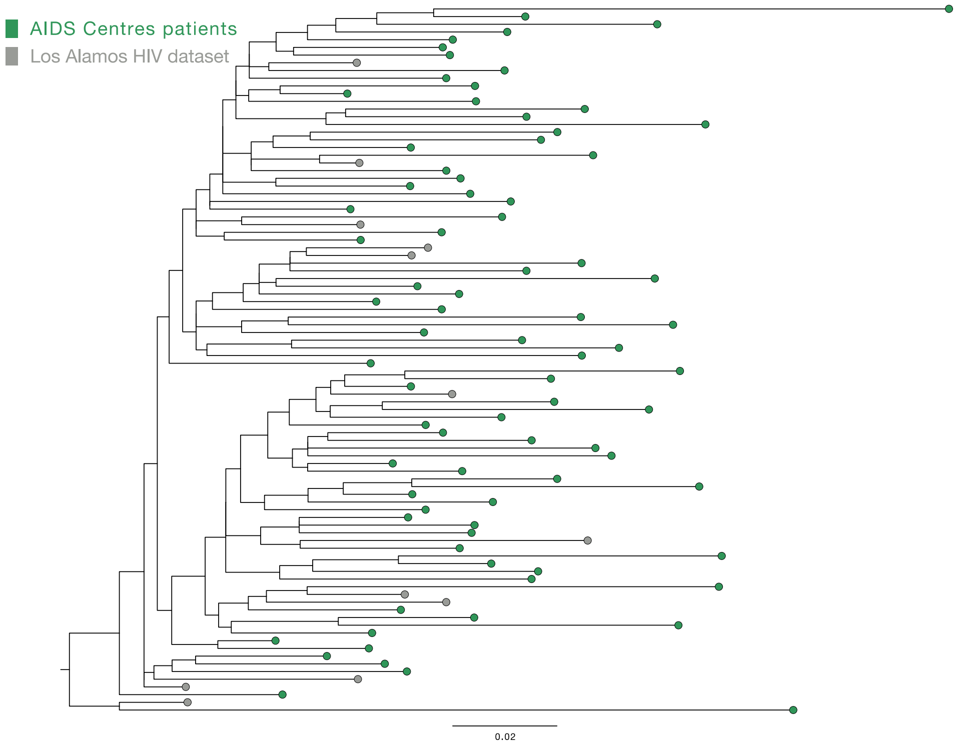

Figure S2. Maximum likelihood phylogeny estimated from the *Kyiv* dataset, mid-point routing.

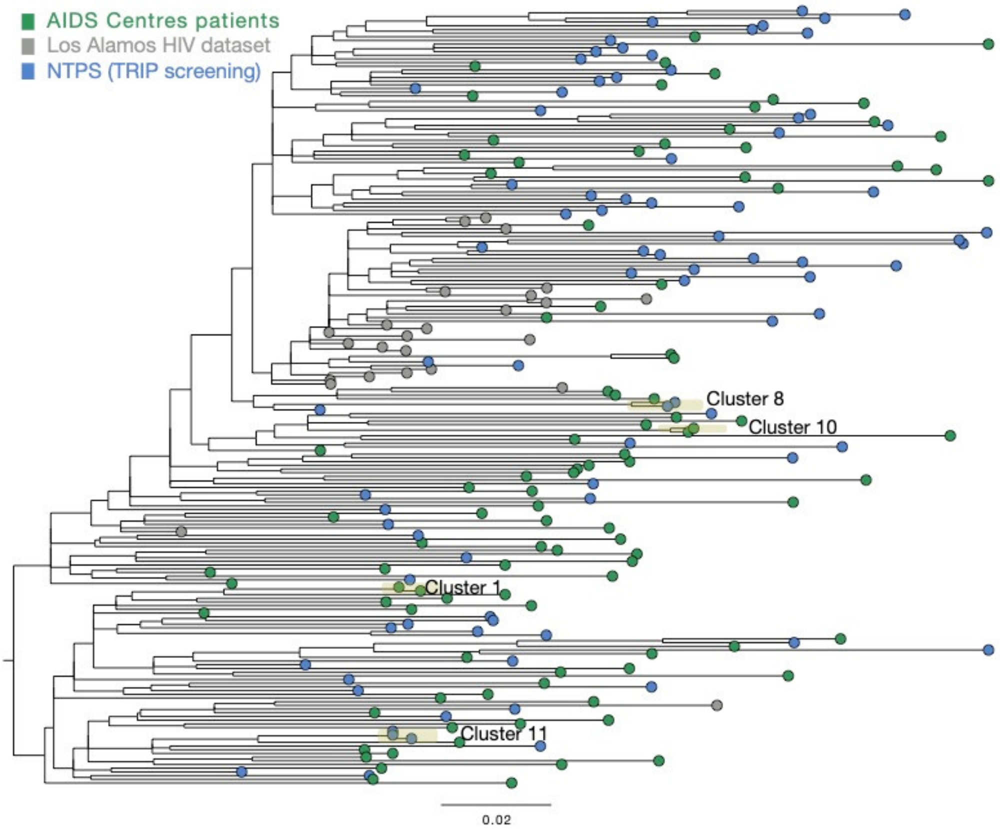

Figure S3. Maximum likelihood phylogeny estimated from the *Odessa* dataset after removing the network-derived sequences, mid-point rooting. Remaining potential transmission clusters are marked in yellow.

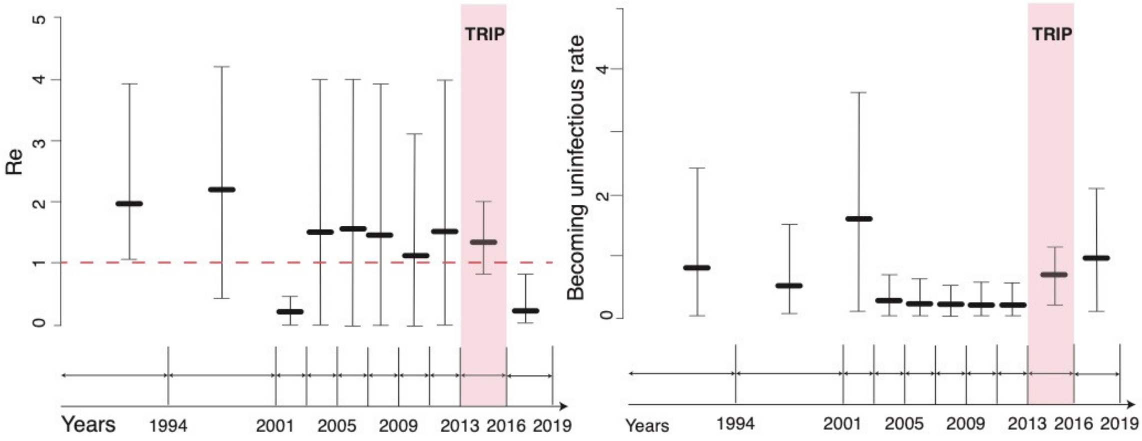

Figure S4. Temporal estimates of the effective reproductive number, R_e, and the becoming uninfectious rate, obtained from the reduced (N=92) *Odessa* dataset. The red dotted line represents the epidemiological threshold of R_e =1

Table S1. Sequence accession numbers

Sequence IDs					
LANL			Newly generated		
AF413987	KY370130	KY370212			
DQ055221	KY370131	KY370218			
DQ055222	KY370132	KY370234			
DQ055223	KY370133	KY370249			
DQ055224	KY370134	KY370251			
DQ055225	KY370136	KY370256			
DQ055226	KY370137	KY370263			
DQ055229	KY370151	KY370269			
DQ055230	KY370152	KY370275			
DQ055231	KY370154	KY370285			
DQ055232	KY370155	KY370375			
DQ055233	KY370156	KY370409			
DQ055234	KY370158	KY370410			
DQ055235	KY370159	KY370411			
DQ055237	KY370160	KY370424			
DQ055238	KY370161	KY370429			
DQ055239	KY370164	KY370433			
DQ055240	KY370168	KY370436			
DQ055241	KY370170	KY370449			
DQ055242	KY370177	KY370452			
DQ823361	KY370178	KY370454			
DQ823365	KY370179	KY370455			
DQ823366	KY370180	KY370457			
DQ823367	KY370181	KY370458			
HQ115067	KY370182	KY370459			
HQ115068	KY370183	KY370463			
HQ115070	KY370184	KY370466			
HQ115071	KY370185	KY370473			
HQ115071	KY370186	KY370474			
HQ115075	KY370190	KY370475			
HQ161906	KY370191	KY370476			
HQ161907	KY370192	KY370477			
HQ161908	KY370194	KY370481			
HQ161908	KY370196	KY370483			
HQ161909	KY370197	KY370488			
HQ161909	KY370393	KY370490			
HQ161910	KY370414	KY370491			
KY370108	KY370415	KY370492			
KY370112	KY370416	KY370494			

KY370113	KY370417	KY370495			
KY370114	KY370418	KY370497			
KY370118	KY370419	KY370504			
KY370121	KY370437	KY370505			
KY370122	KY370107	KY370509			
KY370123	KY370110	KY370510			
KY370124	KY370115	KY370511			
KY370126	KY370120	KY370515			
KY370127	KY370135	KY370520			
KY370128	KY370163	KY370523			
KY370129	KY370189	KY370530			

Table S2. Model selection procedure with the path-sampling (PS) and stepping-stone (SS) marginal likelihood estimators (MLE). A lognormal relaxed molecular clock model was used for each of the three demographic models compared.

Demographic model	MLE	
	PS	SS
Constant population size	-20605.05276	-20607.72465
Exponencial growth population size	-20391.7385	-20396.86683
**Bayesian Skyline Plot**	**-20342.32361**	**-20348.38119**
